# Crystal structure of 3-(4-meth­oxy­phen­yl)-2,3-di­hydro-1*H*-naphtho­[2,1-*b*]pyran-1-one

**DOI:** 10.1107/S2056989015007082

**Published:** 2015-04-22

**Authors:** R. Vasanthi, D. Reuben Jonathan, K. S. Elizhlarasi, B. K. Revathi, G. Usha

**Affiliations:** a PG and Research Department of Physics, Queen Mary’s College, Chennai-4, Tamilnadu, India; b Department of Chemistry, Madras Christian College, Chennai-59, India

**Keywords:** crystal structure, hydro­pyran, flavone derivative

## Abstract

In the title compound, C_20_H_16_O_3_, the hydro­pyran ring adopts a distorted half-chair conformation with the methine C atom and the ring O atom displaced by −0.554 (2) and 0.158 (1) Å, respectively, from the plane of the other four atoms (r.m.s. deviation = 0.020 Å). Its mean plane (all atoms) is inclined to the naphthalene ring system at a dihedral angle of 11.67 (1)°. The dihedral angle between the napthalene ring system and the phenyl ring is 71.84 (1)°. In the crystal, no diectional inter­actions beyond van der Waals contacts could be identified.

## Related literature   

For the biological activity of flavone derivatives, see: Thomas *et al.* (2013 ▸); Kumar *et al.* (2014 ▸); Lee *et al.* (2014 ▸). For further synthetic details, see: Vasanthi *et al.* (2014 ▸).
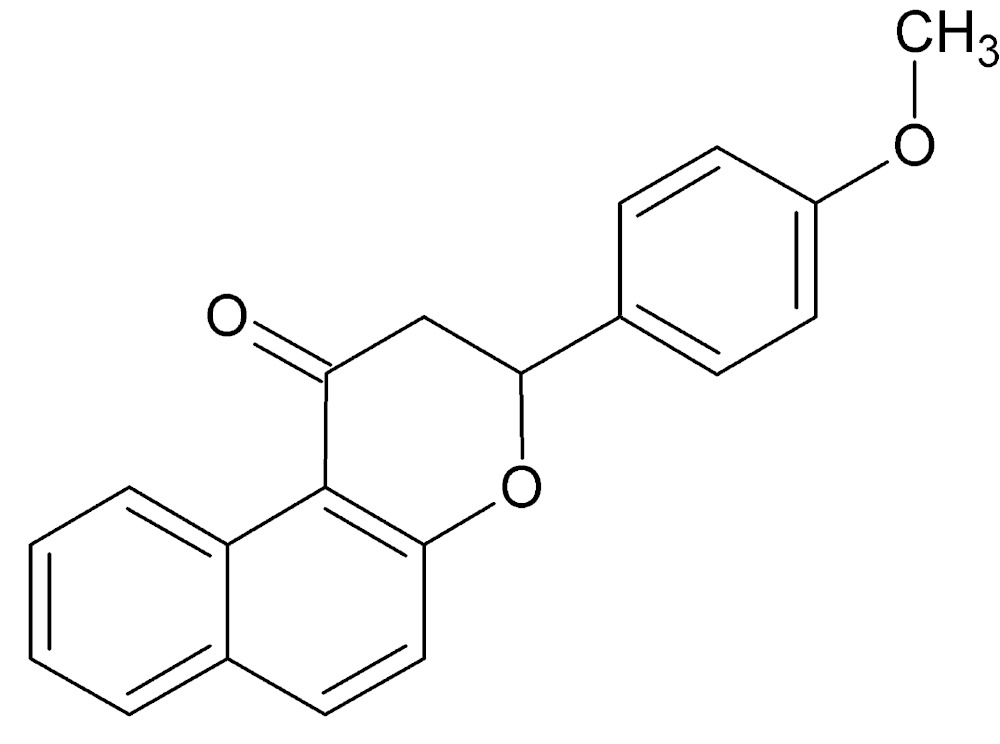


## Experimental   

### Crystal data  

C_20_H_16_O_3_
*M* *_r_* = 304.33
Monoclinic, 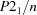

*a* = 7.3612 (3) Å
*b* = 17.8540 (9) Å
*c* = 11.9465 (6) Å
β = 105.697 (2)°
*V* = 1511.54 (12) Å^3^
*Z* = 4
Mo *K*α radiation
μ = 0.09 mm^−1^
*T* = 293 K
0.30 × 0.25 × 0.20 mm

### Data collection  

Bruker Kappa APEXII CCD diffractometer
Absorption correction: multi-scan (*SADABS*; Bruker, 2004 ▸) *T*_min_ = 0.974, *T*_max_ = 0.982
33755 measured reflections
3548 independent reflections
2297 reflections with *I* > 2σ(*I*)
*R*_int_ = 0.035

### Refinement  

*R*[*F* ^2^ > 2σ(*F* ^2^)] = 0.048
*wR*(*F* ^2^) = 0.171
*S* = 1.00
3548 reflections
209 parameters
H-atom parameters constrained
Δρ_max_ = 0.35 e Å^−3^
Δρ_min_ = −0.21 e Å^−3^

### 

Data collection: *APEX2* (Bruker, 2004 ▸); cell refinement: *SAINT* (Bruker, 2004 ▸); data reduction: *SAINT*; program(s) used to solve structure: *SHELXS97* (Sheldrick, 2008 ▸); program(s) used to refine structure: *SHELXL97* (Sheldrick, 2008 ▸); molecular graphics: *ORTEP-3 for Windows* (Farrugia, 2012 ▸); software used to prepare material for publication: *SHELXL97*.

## Supplementary Material

Crystal structure: contains datablock(s) I, New_Global_Publ_Block. DOI: 10.1107/S2056989015007082/hb7401sup1.cif

Structure factors: contains datablock(s) I. DOI: 10.1107/S2056989015007082/hb7401Isup2.hkl

Supporting information file. DOI: 10.1107/S2056989015007082/hb7401Isup3.cml

**Fig. 1.**
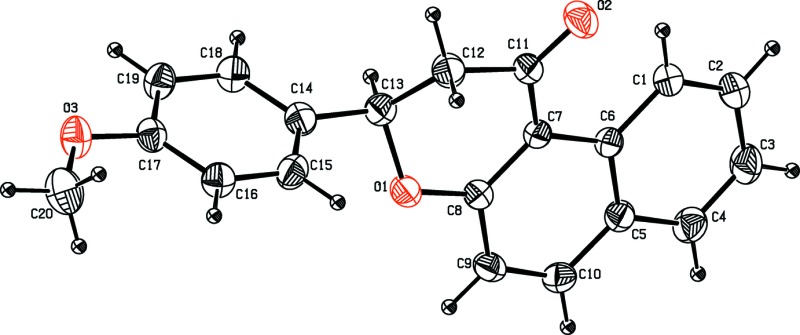
The molecular structure of the title compound, with displacement ellipsoids drawn at the 30% probability level.

. DOI: 10.1107/S2056989015007082/hb7401fig1.tif
The mol­ecular structure of the title compound, with displacement ellipsoids drawn at the 30% probability level.

**Fig. 2.**
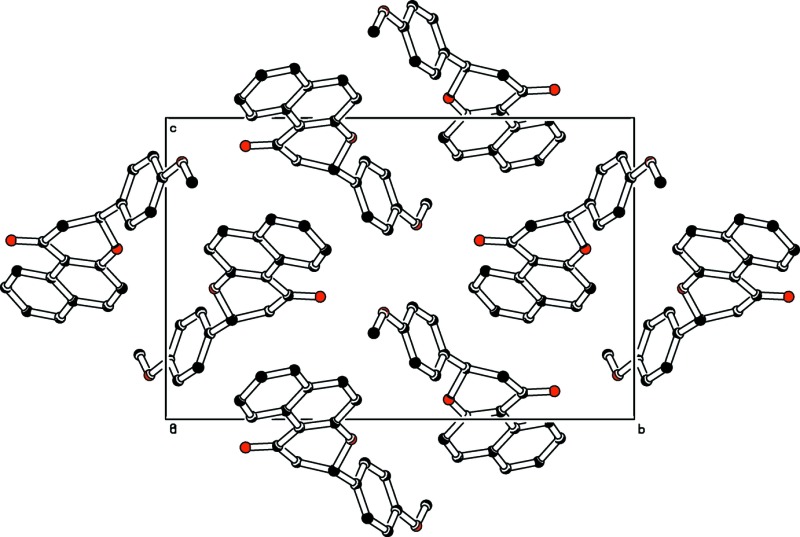
The packing of the molecules in the crystal structure. The dashed lines indicate the hydrogen bonds.

. DOI: 10.1107/S2056989015007082/hb7401fig2.tif
The packing of the mol­ecules in the crystal structure. The dashed lines indicate the hydrogen bonds.

CCDC reference: 1058707

Additional supporting information:  crystallographic information; 3D view; checkCIF report

## supplementary crystallographic information

### S1. Synthesis and crystallization

The procedure (Vasanthi *et al.*, 2014) adopted in the synthesis of the compound 3-(4-meth­oxy­phenyl)-2,3-di­hydro-1H-benzo(f)chromen-1-one is represented here. In a 250 ml round-bottomed flask 2-hy­droxy-1-aceto­naphthone (0.05 mol) and 4-meth­oxy­oxybenzaldehyde (0.05mol) were placed to which about 100 ml of absolute alcohol was added and stirred at room temperature for a time span of 5 minutes. Then about 10 ml of 40% sodium hydroxide solution was added and the mixture was stirred for 6 hours. On adding ice cold water a precipitate was generated which was filtered, washed with sufficient qu­antity of distilled water and dried. The crude product was recrystallized twice from chloro­form (yield = 86%).

### S2. Refinement

Crystal data, data collection and structure refinement details are summarized in Table 1. H atoms were positioned geometrically and treated as riding on their parent atoms, with C—H distance of 0.93-0.97Å with Uiso(H)= 1.5 Ueq(c-methyl) and Uiso(H)= 1.2Ueq(C) for other H atom.

### Crystal data

**Table:**

C_20_H_16_O_3_	*F*(000) = 640
*M**_r_* = 304.33	*D*_x_ = 1.337 Mg m^−^^3^
Monoclinic, *P*2_1_/*n*	Mo *K*α radiation, λ = 0.71073 Å
Hall symbol: -p 2yn	Cell parameters from 3548 reflections
*a* = 7.3612 (3) Å	θ = 2.1–27.7°
*b* = 17.8540 (9) Å	µ = 0.09 mm^−^^1^
*c* = 11.9465 (6) Å	*T* = 293 K
β = 105.697 (2)°	Block, pale yellow
*V* = 1511.54 (12) Å^3^	0.30 × 0.25 × 0.20 mm
*Z* = 4	

### Data collection

**Table:**

Bruker Kappa APEXII CCD diffractometer	3548 independent reflections
Radiation source: fine-focus sealed tube	2297 reflections with *I* > 2σ(*I*)
Graphite monochromator	*R*_int_ = 0.035
ω and φ scan	θ_max_ = 27.7°, θ_min_ = 2.1°
Absorption correction: multi-scan (*SADABS*; Bruker, 2004)	*h* = −9→9
*T*_min_ = 0.974, *T*_max_ = 0.982	*k* = −23→23
33755 measured reflections	*l* = −15→15

### Refinement

**Table:**

Refinement on *F*^2^	Primary atom site location: structure-invariant direct methods
Least-squares matrix: full	Secondary atom site location: difference Fourier map
*R*[*F*^2^ > 2σ(*F*^2^)] = 0.048	Hydrogen site location: inferred from neighbouring sites
*wR*(*F*^2^) = 0.171	H-atom parameters constrained
*S* = 1.00	*w* = 1/[σ^2^(*F*_o_^2^) + (0.0866*P*)^2^ + 0.5483*P*] where *P* = (*F*_o_^2^ + 2*F*_c_^2^)/3
3548 reflections	(Δ/σ)_max_ < 0.001
209 parameters	Δρ_max_ = 0.35 e Å^−^^3^
0 restraints	Δρ_min_ = −0.21 e Å^−^^3^

### Special details

**Table:**

Geometry. All e.s.d.'s (except the e.s.d. in the dihedral angle between two l.s. planes) are estimated using the full covariance matrix. The cell e.s.d.'s are taken into account individually in the estimation of e.s.d.'s in distances, angles and torsion angles; correlations between e.s.d.'s in cell parameters are only used when they are defined by crystal symmetry. An approximate (isotropic) treatment of cell e.s.d.'s is used for estimating e.s.d.'s involving l.s. planes.
Refinement. Refinement of *F*^2^ against ALL reflections. The weighted *R*-factor *wR* and goodness of fit *S* are based on *F*^2^, conventional *R*-factors *R* are based on *F*, with *F* set to zero for negative *F*^2^. The threshold expression of *F*^2^ > σ(*F*^2^) is used only for calculating *R*-factors(gt) *etc*. and is not relevant to the choice of reflections for refinement. *R*-factors based on *F*^2^ are statistically about twice as large as those based on *F*, and *R*- factors based on ALL data will be even larger.

### Fractional atomic coordinates and isotropic or equivalent isotropic displacement parameters (Å^2^)

**Table:**

	*x*	*y*	*z*	*U*_iso_*/*U*_eq_	
C1	0.8210 (3)	0.31670 (11)	0.50857 (17)	0.0479 (5)	
H1	0.7380	0.3483	0.4572	0.057*	
C2	0.9984 (3)	0.34116 (12)	0.5627 (2)	0.0569 (6)	
H2	1.0348	0.3890	0.5468	0.068*	
C3	1.1261 (3)	0.29601 (14)	0.6411 (2)	0.0615 (6)	
H3	1.2457	0.3139	0.6785	0.074*	
C4	1.0749 (3)	0.22571 (13)	0.66264 (19)	0.0552 (5)	
H4	1.1603	0.1956	0.7152	0.066*	
C5	0.8940 (3)	0.19745 (11)	0.60665 (16)	0.0442 (4)	
C6	0.7616 (2)	0.24405 (10)	0.52940 (15)	0.0398 (4)	
C7	0.5772 (2)	0.21474 (10)	0.47367 (14)	0.0380 (4)	
C8	0.5436 (2)	0.13995 (10)	0.48896 (15)	0.0404 (4)	
C9	0.6770 (3)	0.09415 (11)	0.56488 (18)	0.0480 (5)	
H9	0.6500	0.0440	0.5741	0.058*	
C10	0.8442 (3)	0.12317 (11)	0.62434 (17)	0.0494 (5)	
H10	0.9285	0.0935	0.6782	0.059*	
C11	0.4166 (3)	0.26181 (11)	0.41137 (16)	0.0444 (4)	
C12	0.2355 (3)	0.22029 (11)	0.3582 (2)	0.0534 (5)	
H12A	0.1573	0.2214	0.4118	0.064*	
H12B	0.1675	0.2456	0.2875	0.064*	
C13	0.2685 (3)	0.14097 (11)	0.33062 (18)	0.0478 (5)	
H13	0.3405	0.1408	0.2726	0.057*	
C14	0.0947 (3)	0.09419 (11)	0.28505 (18)	0.0461 (5)	
C15	−0.0210 (3)	0.07528 (12)	0.35425 (18)	0.0537 (5)	
H15	0.0094	0.0918	0.4309	0.064*	
C16	−0.1816 (3)	0.03216 (12)	0.31180 (18)	0.0514 (5)	
H16	−0.2587	0.0201	0.3593	0.062*	
C17	−0.2257 (3)	0.00744 (10)	0.19866 (17)	0.0451 (5)	
C18	0.0466 (3)	0.06940 (12)	0.17210 (18)	0.0511 (5)	
H18	0.1226	0.0818	0.1241	0.061*	
C19	−0.1111 (3)	0.02680 (12)	0.12893 (18)	0.0521 (5)	
H19	−0.1413	0.0107	0.0520	0.062*	
C20	−0.5019 (3)	−0.05604 (15)	0.2142 (3)	0.0741 (7)	
H20A	−0.4325	−0.0834	0.2814	0.111*	
H20B	−0.6009	−0.0870	0.1684	0.111*	
H20C	−0.5558	−0.0120	0.2386	0.111*	
O1	0.38066 (18)	0.10428 (7)	0.43445 (12)	0.0496 (4)	
O2	0.4204 (2)	0.32953 (8)	0.40683 (15)	0.0635 (5)	
O3	−0.3787 (2)	−0.03475 (9)	0.14670 (14)	0.0619 (4)	

### Atomic displacement parameters (Å^2^)

**Table:**

	*U*^11^	*U*^22^	*U*^33^	*U*^12^	*U*^13^	*U*^23^
C1	0.0537 (11)	0.0423 (11)	0.0461 (11)	−0.0051 (8)	0.0107 (9)	−0.0012 (8)
C2	0.0593 (13)	0.0490 (12)	0.0598 (13)	−0.0147 (10)	0.0116 (10)	−0.0031 (10)
C3	0.0483 (11)	0.0647 (14)	0.0659 (14)	−0.0137 (10)	0.0059 (10)	−0.0081 (11)
C4	0.0475 (11)	0.0594 (13)	0.0524 (12)	0.0030 (9)	0.0026 (9)	−0.0006 (10)
C5	0.0453 (10)	0.0439 (10)	0.0416 (10)	0.0005 (8)	0.0083 (8)	−0.0015 (8)
C6	0.0460 (10)	0.0382 (9)	0.0351 (9)	−0.0008 (7)	0.0110 (7)	−0.0026 (7)
C7	0.0438 (9)	0.0356 (9)	0.0336 (9)	0.0003 (7)	0.0089 (7)	−0.0012 (7)
C8	0.0424 (10)	0.0377 (10)	0.0404 (10)	−0.0021 (7)	0.0103 (7)	0.0002 (8)
C9	0.0535 (11)	0.0367 (10)	0.0514 (11)	0.0010 (8)	0.0099 (9)	0.0069 (8)
C10	0.0523 (11)	0.0460 (11)	0.0451 (11)	0.0071 (9)	0.0047 (9)	0.0066 (9)
C11	0.0499 (10)	0.0365 (10)	0.0443 (10)	0.0010 (8)	0.0084 (8)	0.0005 (8)
C12	0.0491 (11)	0.0445 (11)	0.0597 (13)	0.0020 (8)	0.0029 (9)	0.0016 (9)
C13	0.0472 (11)	0.0463 (11)	0.0473 (11)	−0.0005 (8)	0.0084 (8)	0.0003 (9)
C14	0.0424 (10)	0.0417 (10)	0.0520 (11)	0.0004 (8)	0.0091 (8)	−0.0005 (8)
C15	0.0587 (12)	0.0587 (13)	0.0419 (11)	−0.0037 (10)	0.0104 (9)	−0.0084 (9)
C16	0.0515 (11)	0.0538 (12)	0.0512 (12)	−0.0021 (9)	0.0178 (9)	−0.0032 (9)
C17	0.0414 (10)	0.0399 (10)	0.0520 (11)	0.0015 (8)	0.0093 (8)	−0.0043 (8)
C18	0.0495 (11)	0.0545 (12)	0.0518 (12)	−0.0038 (9)	0.0182 (9)	−0.0047 (9)
C19	0.0536 (11)	0.0571 (13)	0.0461 (11)	−0.0038 (9)	0.0146 (9)	−0.0103 (9)
C20	0.0521 (13)	0.0760 (17)	0.098 (2)	−0.0161 (12)	0.0263 (13)	−0.0099 (15)
O1	0.0474 (8)	0.0395 (7)	0.0554 (8)	−0.0048 (6)	0.0028 (6)	0.0053 (6)
O2	0.0619 (10)	0.0377 (8)	0.0802 (11)	0.0039 (6)	0.0008 (8)	0.0028 (7)
O3	0.0538 (9)	0.0633 (10)	0.0681 (10)	−0.0171 (7)	0.0159 (7)	−0.0162 (8)

### Geometric parameters (Å, º)

**Table:**

C1—C2	1.362 (3)	C12—C13	1.489 (3)
C1—C6	1.413 (3)	C12—H12A	0.9700
C1—H1	0.9300	C12—H12B	0.9700
C2—C3	1.391 (3)	C13—O1	1.448 (2)
C2—H2	0.9300	C13—C14	1.501 (3)
C3—C4	1.355 (3)	C13—H13	0.9800
C3—H3	0.9300	C14—C18	1.372 (3)
C4—C5	1.412 (3)	C14—C15	1.380 (3)
C4—H4	0.9300	C15—C16	1.387 (3)
C5—C10	1.407 (3)	C15—H15	0.9300
C5—C6	1.417 (3)	C16—C17	1.375 (3)
C6—C7	1.439 (2)	C16—H16	0.9300
C7—C8	1.379 (2)	C17—O3	1.357 (2)
C7—C11	1.477 (2)	C17—C19	1.381 (3)
C8—O1	1.359 (2)	C18—C19	1.366 (3)
C8—C9	1.405 (3)	C18—H18	0.9300
C9—C10	1.347 (3)	C19—H19	0.9300
C9—H9	0.9300	C20—O3	1.419 (3)
C10—H10	0.9300	C20—H20A	0.9600
C11—O2	1.211 (2)	C20—H20B	0.9600
C11—C12	1.507 (3)	C20—H20C	0.9600
			
C2—C1—C6	120.91 (19)	C13—C12—H12B	109.1
C2—C1—H1	119.5	C11—C12—H12B	109.1
C6—C1—H1	119.5	H12A—C12—H12B	107.8
C1—C2—C3	121.3 (2)	O1—C13—C12	109.26 (16)
C1—C2—H2	119.3	O1—C13—C14	106.95 (15)
C3—C2—H2	119.3	C12—C13—C14	115.83 (16)
C4—C3—C2	119.52 (19)	O1—C13—H13	108.2
C4—C3—H3	120.2	C12—C13—H13	108.2
C2—C3—H3	120.2	C14—C13—H13	108.2
C3—C4—C5	121.08 (19)	C18—C14—C15	118.26 (18)
C3—C4—H4	119.5	C18—C14—C13	120.25 (18)
C5—C4—H4	119.5	C15—C14—C13	121.49 (18)
C10—C5—C4	121.09 (18)	C14—C15—C16	121.34 (19)
C10—C5—C6	119.34 (17)	C14—C15—H15	119.3
C4—C5—C6	119.56 (18)	C16—C15—H15	119.3
C1—C6—C5	117.55 (17)	C17—C16—C15	119.26 (19)
C1—C6—C7	123.44 (17)	C17—C16—H16	120.4
C5—C6—C7	118.98 (16)	C15—C16—H16	120.4
C8—C7—C6	118.11 (16)	O3—C17—C16	125.00 (19)
C8—C7—C11	118.04 (16)	O3—C17—C19	115.47 (18)
C6—C7—C11	123.53 (16)	C16—C17—C19	119.52 (18)
O1—C8—C7	123.85 (16)	C19—C18—C14	121.16 (19)
O1—C8—C9	114.13 (16)	C19—C18—H18	119.4
C7—C8—C9	122.01 (17)	C14—C18—H18	119.4
C10—C9—C8	119.73 (18)	C18—C19—C17	120.47 (19)
C10—C9—H9	120.1	C18—C19—H19	119.8
C8—C9—H9	120.1	C17—C19—H19	119.8
C9—C10—C5	121.42 (17)	O3—C20—H20A	109.5
C9—C10—H10	119.3	O3—C20—H20B	109.5
C5—C10—H10	119.3	H20A—C20—H20B	109.5
O2—C11—C7	124.46 (17)	O3—C20—H20C	109.5
O2—C11—C12	120.04 (17)	H20A—C20—H20C	109.5
C7—C11—C12	115.37 (16)	H20B—C20—H20C	109.5
C13—C12—C11	112.50 (16)	C8—O1—C13	115.06 (14)
C13—C12—H12A	109.1	C17—O3—C20	117.87 (18)
C11—C12—H12A	109.1		
			
C6—C1—C2—C3	−0.8 (3)	C8—C7—C11—C12	−7.0 (2)
C1—C2—C3—C4	1.3 (4)	C6—C7—C11—C12	179.61 (17)
C2—C3—C4—C5	0.1 (4)	O2—C11—C12—C13	157.9 (2)
C3—C4—C5—C10	176.6 (2)	C7—C11—C12—C13	−26.1 (2)
C3—C4—C5—C6	−2.0 (3)	C11—C12—C13—O1	55.1 (2)
C2—C1—C6—C5	−1.1 (3)	C11—C12—C13—C14	175.91 (17)
C2—C1—C6—C7	−179.08 (19)	O1—C13—C14—C18	−126.0 (2)
C10—C5—C6—C1	−176.18 (18)	C12—C13—C14—C18	112.0 (2)
C4—C5—C6—C1	2.5 (3)	O1—C13—C14—C15	54.4 (2)
C10—C5—C6—C7	1.9 (3)	C12—C13—C14—C15	−67.7 (3)
C4—C5—C6—C7	−179.48 (17)	C18—C14—C15—C16	0.2 (3)
C1—C6—C7—C8	171.55 (18)	C13—C14—C15—C16	179.89 (18)
C5—C6—C7—C8	−6.4 (3)	C14—C15—C16—C17	0.4 (3)
C1—C6—C7—C11	−15.1 (3)	C15—C16—C17—O3	−179.74 (19)
C5—C6—C7—C11	167.02 (17)	C15—C16—C17—C19	−0.9 (3)
C6—C7—C8—O1	−175.03 (16)	C15—C14—C18—C19	−0.3 (3)
C11—C7—C8—O1	11.2 (3)	C13—C14—C18—C19	−179.97 (19)
C6—C7—C8—C9	5.9 (3)	C14—C18—C19—C17	−0.2 (3)
C11—C7—C8—C9	−167.86 (18)	O3—C17—C19—C18	179.78 (18)
O1—C8—C9—C10	−179.81 (18)	C16—C17—C19—C18	0.9 (3)
C7—C8—C9—C10	−0.7 (3)	C7—C8—O1—C13	20.1 (3)
C8—C9—C10—C5	−4.2 (3)	C9—C8—O1—C13	−160.76 (17)
C4—C5—C10—C9	−175.1 (2)	C12—C13—O1—C8	−52.9 (2)
C6—C5—C10—C9	3.5 (3)	C14—C13—O1—C8	−178.94 (15)
C8—C7—C11—O2	168.8 (2)	C16—C17—O3—C20	−0.9 (3)
C6—C7—C11—O2	−4.6 (3)	C19—C17—O3—C20	−179.7 (2)
