# Supplementary figures and images for: Crystal structure of 3-(4-meth­oxy­phen­yl)-2,3-di­hydro-1H-naphtho­[2,1-b]pyran-1-one
Source: Acta Crystallogr E Crystallogr Commun. 2015 Apr 22;71(Pt 5):o332. doi: 10.1107/S2056989015007082 (PMC4420115; doi:10.1107/S2056989015007082)

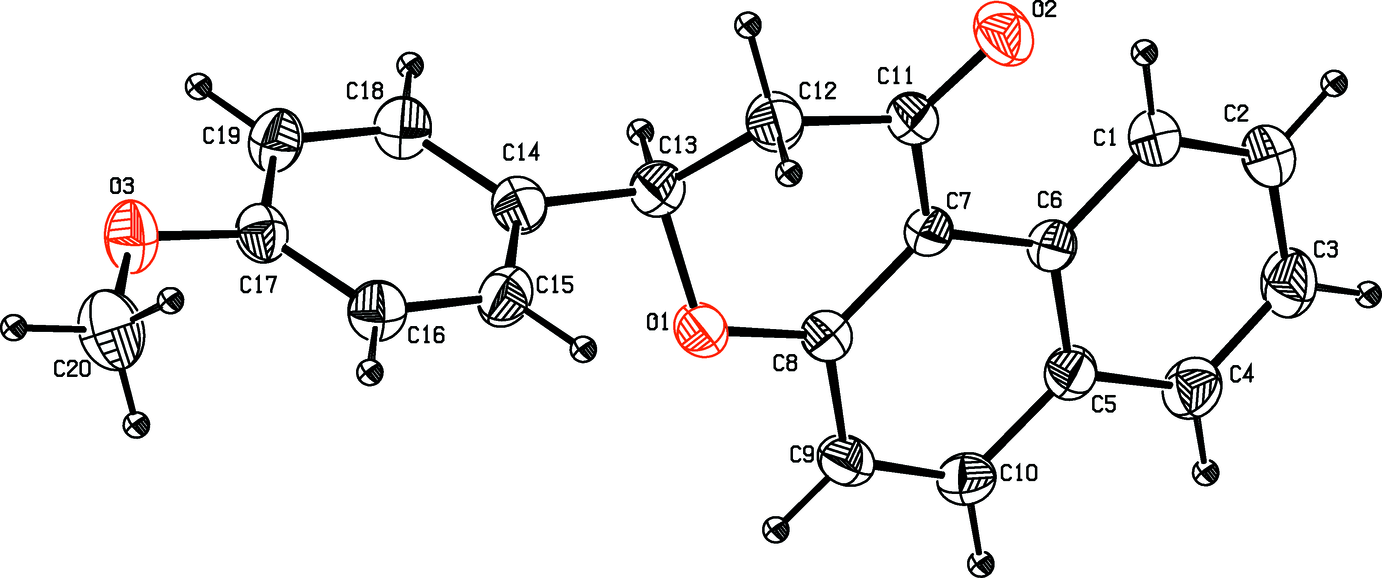

Supplement: Supplementary file 4 [file e-71-0o332-fig1.tif]

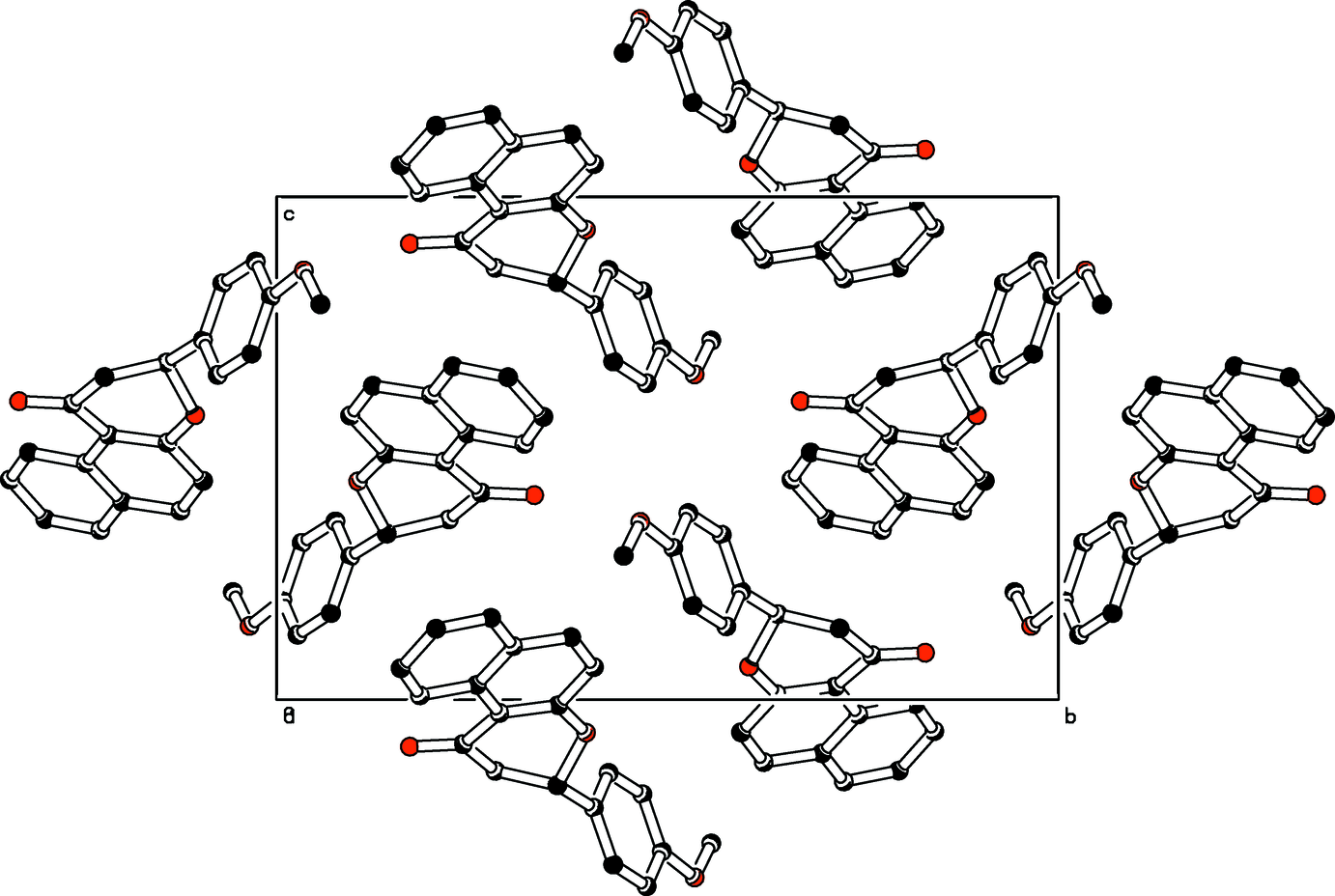

Supplement: Supplementary file 5 [file e-71-0o332-fig2.tif]
